# Venetoclax-Resistant T-ALL Cells Display Distinct Cancer Stem Cell Signatures and Enrichment of Cytokine Signaling

**DOI:** 10.3390/ijms24055004

**Published:** 2023-03-05

**Authors:** Kinjal Shah, Lina Al Ashiri, Ahmad Nasimian, Mehreen Ahmed, Julhash U. Kazi

**Affiliations:** 1 Division of Translational Cancer Research, Department of Laboratory Medicine, Lund University, 22381 Lund, Sweden; 2 Lund Stem Cell Center, Department of Laboratory Medicine, Lund University, 22184 Lund, Sweden

**Keywords:** apoptosis resistance, targeted therapy, leukemia, BCL2 inhibition, navitoclax

## Abstract

Therapy resistance remains one of the major challenges for cancer treatment that largely limits treatment benefits and patient survival. The underlying mechanisms that lead to therapy resistance are highly complicated because of the specificity to the cancer subtype and therapy. The expression of the anti-apoptotic protein BCL2 has been shown to be deregulated in T-cell acute lymphoblastic leukemia (T-ALL), where different T-ALL cells display a differential response to the BCL2-specific inhibitor venetoclax. In this study, we observed that the expression of anti-apoptotic BCL2 family genes, such as *BCL2*, *BCL2L1*, and *MCL1*, is highly varied in T-ALL patients, and inhibitors targeting proteins coded by these genes display differential responses in T-ALL cell lines. Three T-ALL cell lines (ALL-SIL, MOLT-16, and LOUCY) were highly sensitive to BCL2 inhibition within a panel of cell lines tested. These cell lines displayed differential *BCL2* and *BCL2L1* expression. Prolonged exposure to venetoclax led to the development of resistance to it in all three sensitive cell lines. To understand how cells developed venetoclax resistance, we monitored the expression of *BCL2*, *BCL2L1*, and *MCL1* over the treatment period and compared gene expression between resistant cells and parental sensitive cells. We observed a different trend of regulation in terms of BCL2 family gene expression and global gene expression profile including genes reported to be expressed in cancer stem cells. Gene set enrichment analysis (GSEA) showed enrichment of cytokine signaling in all three cell lines which was supported by the phospho-kinase array where STAT5 phosphorylation was found to be elevated in resistant cells. Collectively, our data suggest that venetoclax resistance can be mediated through the enrichment of distinct gene signatures and cytokine signaling pathways.

## 1. Introduction

T-cell acute lymphoblastic leukemia (T-ALL) is a type of acute leukemia that develops from immature white blood cells [1]. It is considered to be one of the most aggressive forms of leukemia, and it occurs in both children and adults representing around 15% and 25% of the patients, respectively [2]. T-ALL is characterized by several unique genetic features that disrupt key signaling pathways, including the abnormal activation of NOTCH signaling, deregulated expression of transcription factors and tumor suppressors, abnormal activation of kinase and cytokine signaling, and disruption of cell cycle regulation [1,3,4,5]. At present, a majority of T-ALL patients are treated with chemotherapy [6]. Despite the fact that survival rates have improved in children with T-ALL, as a result of better risk assessment and chemotherapy regimens, the disease is still very hard to treat upon relapse, and there are few treatment options available [7]. This highlights the need for new therapies to better treat this patient population.

The normal development of T-cells is a tightly controlled process [8]. Among the various regulators, the expression of B-cell lymphoma 2 (BCL2) family proteins plays an important role in this process. For example, BCL2 expression is upregulated in double-negative thymocytes, then downregulated in a majority of double-positive thymocytes, and finally upregulated in mature single-positive thymocytes [9,10]. Expression of this family of proteins also varies in T-ALL. A group of T-ALL patients display a higher level of BCL2 expression and therefore show sensitivity to the BCL2-specific inhibitor venetoclax [11,12,13,14].

The BCL2 family proteins are characterized by the presence of a highly conserved BH3 domain. These family proteins are subdivided into three groups: multidomain proapoptotic (BAK [*BCL2L7*], BAX [*BCL2L4*], BOK [*BCL2L9*]), multidomain antiapoptotic (BCL2, BCL-B [*BCL2L10*], BCL-W [*BCL2L2*], BCL-XL [*BCL2L1*], BFL-1 [*BCL2A1* or *BCL2L5*], MCL-1 [*BCL2L3*]), and BH3-only proteins, including the BH3-only activators (BID [*BCL2L11*], BIM [*BCL2L11*], PUMA [*BBC3*]) and BH3-only sensitizers (BAD [*BCL2L*8], BCL-G [*BCL2L14*], BCL-RAMBO [*BCL2L13*], BIK, BMF, HRK, NOXA [*PMAIP1*], SPIKE) [15,16]. In response to cellular stress, such as DNA damage, energy stress, growth factor withdrawal, hypoxia, etc., the expression of BH3-only members is elevated, and, therefore, proapoptotic members are activated through the release of antiapoptotic members or by the binding of activators. These facilitate the oligomerization of proapoptotic members, thereby creating channels in the mitochondrial outer membrane to release Cytochrome c. Thus, the expression levels of BCL2 family proteins determine whether the cell will go into apoptosis or not upon stress [16]. 

Since the BH3 domain plays an important role in the regulation of BCL2 family proteins, an initial attempt was taken to inhibit BCL2 by BH3 mimetics. Several BH3 mimetics (obatoclax, ABT737, sabutoclax, and navitoclax) were reported to be non-specific, and displayed higher toxicity [17,18,19,20,21,22,23]. The BCL2-specific inhibitor venetoclax was shown to be highly specific and well-tolerated and received FDA approval for the treatment of certain indications of chronic lymphoblastic leukemia and acute myeloid leukemia [21]. Venetoclax binds to BCL2 and interrupts association with BH3-only activators such as BIM, leading to the activation of the proapoptotic protein BAX [21]. Although venetoclax specifically inhibits BCL2, sensitivity varies from patient to patient and cannot always be explained by the level of BCL2 expression [14,24,25]. The expression of several other BCL2 family members, such as BCL-XL and MCL1, can play a role in venetoclax sensitivity, and inhibitors targeting multiple proteins displayed better efficacy with a cost of higher toxicity [25,26,27]. Elevated expression of those genes was reported to be maintained by secreted proteins such as IL10 and CD154, activation of toll-like receptor 9 (TLR9), and NFκB signaling [21,28]. Furthermore, copy number alterations in *TP53*, *SF3B1, RB1, NOTCH1, VD274,* and *BRAF*, mutations in *BCL2*, and ERK1/2-mediated phosphorylation of BIM contributes to venetoclax resistance [21,28,29,30,31] Although substantial progress has been made, a deeper understanding of resistance to BCL2 inhibitors is required in order to create more effective and safer treatments.

In this study, we generated venetoclax-resistant cell lines via prolonged exposure to increasing concentrations of venetoclax, to study the underlying mechanisms of its resistance. We used RNAseq to determine the deregulated gene signatures in resistant cells.

## 2. Results

### 2.1. Pro-Survival BCL2 Family Members Display Differential Expression in T-ALL

The expression level of BCL2 varies during T-cell development, which is maintained in different subgroups of T-ALL. To explore the expression pattern of pro-survival BCL2 family proteins, we analyzed the mRNA expression of *BCL2*, *BCL2L1* (BCL-XL), *BCL2L2* (BCL-W), and *MCL-1* from seven T-ALL patient cohorts in which a wide range of variations in terms of their expression was demonstrated (Supplementary Figure S1A,B). *MCL-1* displayed consistently higher expression, with a moderate variation in expression (variance between 0.7 and 1.75), except in the GSE28703 cohort. *BCL2L2* expression was comparatively low, with lower expression variation (variance between 0.05 and 0.93). Expression of *BCL2L1* and *BCL2* was different in different cohorts, and the variance of *BCL2* was consistently high (variance between 1.69 and 3.44) in all cohorts except for a pediatric T-ALL cohort, GSE26713 (variance 0.61). However, we did not see any correlation between the expression of these four genes. Because higher variation in *BCL2* expression could be explained by the presence of early T-cell precursor (ETP)-ALL, we analyzed three patient cohorts (in which patients were labeled as ETP- or non-ETP-ALL) with the ETP-ALL samples removed. Although the variance was reduced slightly, it remained higher for *BCL2* compared to the other three genes (Supplementary Figure S1C,D), suggesting that *BCL2* expression could be increased in non-ETP ALL patients.

**Figure 1:**
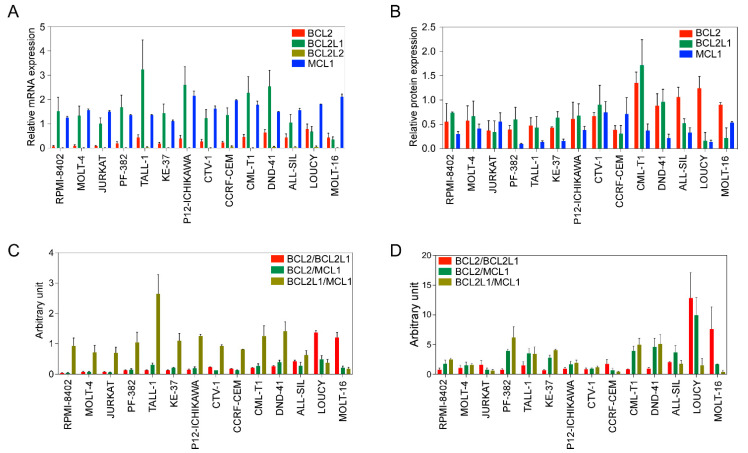
BCL2 family expression varies in T-ALL cell lines. (**A**) Total mRNA from different cell lines was collected. Expression of *BCL2, BCL2L1, BCL2L2*, and *MCL1* was measured via qPCR. (**B**) Protein expression of BCL2, BCL2L1 and MCL1 was determined via western blots using specific antibodies. Band intensities were quantified using ImageJ. (**C**) Relative mRNA expression determined via qPCR was used to calculate the ratio. (**D**) Protein expression values determined in (**B**) were used to calculate the ratio. All experiments were repeated three times and the error bars represent SEM.

Next, we used a panel of cell lines (Supplementary Table S1) to further assess the expression of antiapoptotic BCL2 family members. The expression of BCL2, BCL2L1, and MCL1 was detected at the mRNA (Figure 1A) and protein levels (Figure 1B) in all cell lines. *BCL2L2* expression was relatively low-to-undetectable and the expression of *MCL1* was higher in all cell lines (Figure 1A). *BCL2* expression was lower in RPMI-8402, MOLT-4, and JURKAT cell lines, while other cell lines displayed almost equal levels of expression. *BCL2L1* expression was lower in LOUCY and MOLT-16 cells but higher in all other cell lines.

Because the *BCL2/BCL2L1* ratio can influence the sensitivity of BCL2-specific inhibitors [25], we next determined the ratio between *BCL2/BCL2L1*, as well as *BCL2/MCL1* and *BCL2L1/MCL1*. While the *BCL2L1/MCL1* ratio was high in several cell lines, the *BCL2/BCL2L1* ratio was only higher in the LOUCY and MOLT-16 cell lines, and the *BCL2/MCL1* ratio was almost the same in all cell lines (Figure 1C). Similar to the mRNA ratio, the BCL2/BCL2L1 protein ratio was higher in LOUCY and MOLT-16 cells (Figure 1D). Collectively, our data suggest that the expression of BCL2 family proteins varies among T-ALL patients as well as among T-ALL cell lines.

### 2.2. T-ALL Cells Exhibit a Differential Response to the Inhibition of BCL2 Family Members

**Figure 2:**
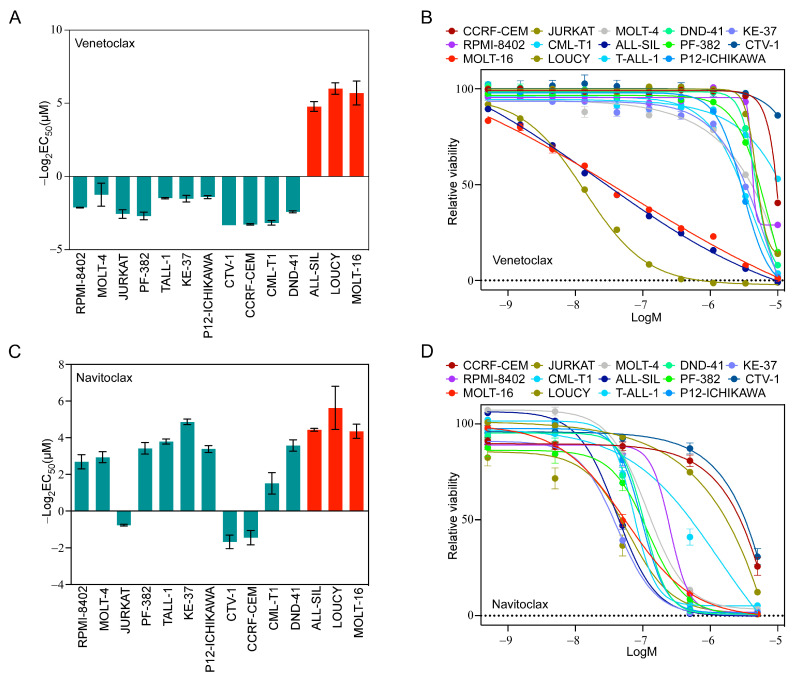
T-ALL cells display differential sensitivity to venetoclax and navitoclax. T-ALL cell lines were treated with different concentrations of (**A**,**B**) venetoclax and (**C**,**D**) navitoclax for 48 h. Cell viability was measured using PrestoBlue cell viability assay and GraphPad Prism was used to calculate EC_50_. Error bars represent SEM.

Because we observed a higher ratio of BCL2/BCL2L1 in LOUCY and MOLT-16 cells, we hypothesized that LOUCY and MOLT-16 would display higher sensitivity to a BCL2-specific inhibitor, while others would display resistance. As anticipated, LOUCY and MOLT-16 cells were highly sensitive to venetoclax and several other cell lines did not respond (Figure 2A,B). Although ALL-SIL displayed a low BCL2/BCL2L1 ratio (Supplementary Figure S2A), as opposed to LOUCY and MOLT-16, it was also highly sensitive (Figure 2A,B). Therefore, it is likely that the BCL2/BCL2L1 ratio cannot reliably predict venetoclax sensitivity. As we observed a higher expression of BCL2L1 in several cell lines, we used navitoclax, which inhibits BCL2, BCL2L1, and BCL2L2. The majority of cell lines were sensitive to navitoclax (Figure 2C,D), and the response was largely but not completely dependent on the BCL2L1/MCL-1 ratio. Similarly, inhibitor data from the Genomics of Drug Sensitivity in Cancer (GDSC) cell line project also displayed wide variation in the response in T-ALL cell lines (Supplementary Figure S2B–E). Taken together, our data suggest that the BCL2/BCL2L1 and BCL2L1/MCL-1 ratios have limited applications in the prediction of venetoclax and navitoclax sensitivity, respectively.

### 2.3. Prolonged Venetoclax Treatment Differentially Regulates BCL2 Expression

**Figure 3:**
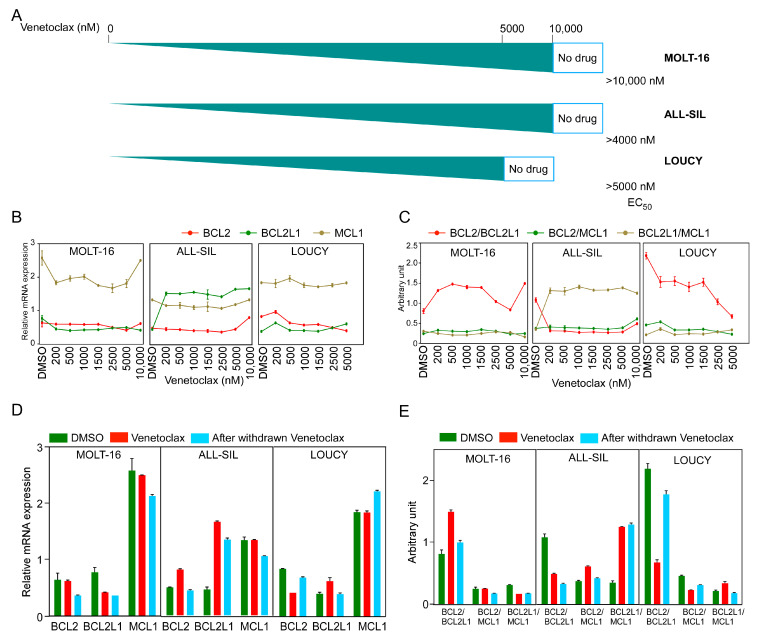
Generation of venetoclax-resistant MOLT-16, ALL-SIL, and LOUCY cells. T-ALL cell lines were treated with increasing concentrations of venetoclax (from 10 nM to 10 µM). (**A**) Schematic representation of the generation of venetoclax-resistant cells. EC_50_ values (nM) were measured using the PrestoBlue cell viability assay. (**B**,**C**) Relative mRNA expression and ratios of *BCL2*, *BCL2L1*, and *MCL1* were plotted during the generation of venetoclax-resistant cells. Error bars represent SEM. (**D**,**E**) Relative mRNA expression and ratios from three cell lines before and after venetoclax resistance, in the presence or absence of venetoclax in culture medium and after withdrawal of venetoclax from the culture medium.

To understand how cells develop venetoclax resistance, we treated all three sensitive cell lines with increasing concentrations of venetoclax until cells developed resistance to at least 5 µM venetoclax (Figure 3A). We measured *BCL2*, *BCL2L1*, and *MCL-1* expression by sampling at different venetoclax concentrations and observed differential regulation during treatment (Figure 3B). While the MOLT-16 and LOUCY cell lines displayed a similar expression pattern of all three genes, *BCL2L1* expression was consistently high in venetoclax-treated ALL-SIL cells. The *BCL2/BCL2L1* ratio was increased in MOLT-16 cells but decreased in ALL-SIL and LOUCY cell lines (Figure 3C). In ALL-SIL, the ratio was decreased in cells at the beginning of the treatment and stayed low during the treatment period. Upon withdrawal of venetoclax for two weeks, expression of both *BCL2* and *BCL2L1* was decreased in MOLT-16 and ALL-SIL cells, while *BCL2* expression was increased in LOUCY cells (Figure 3D and Supplementary Figure S3). The *BCL2/BCL2L1* ratio was restored to the initial value in MOLT-16 and LOUCY cells but reduced in ALL-SIL (Figure 3E), further demonstrating that the *BCL2/BCL2L1* ratio has limited applicability for venetoclax response prediction. 

### 2.4. Venetoclax Resistant Cells Displayed Enrichment of Different Pathways

**Figure 4:**
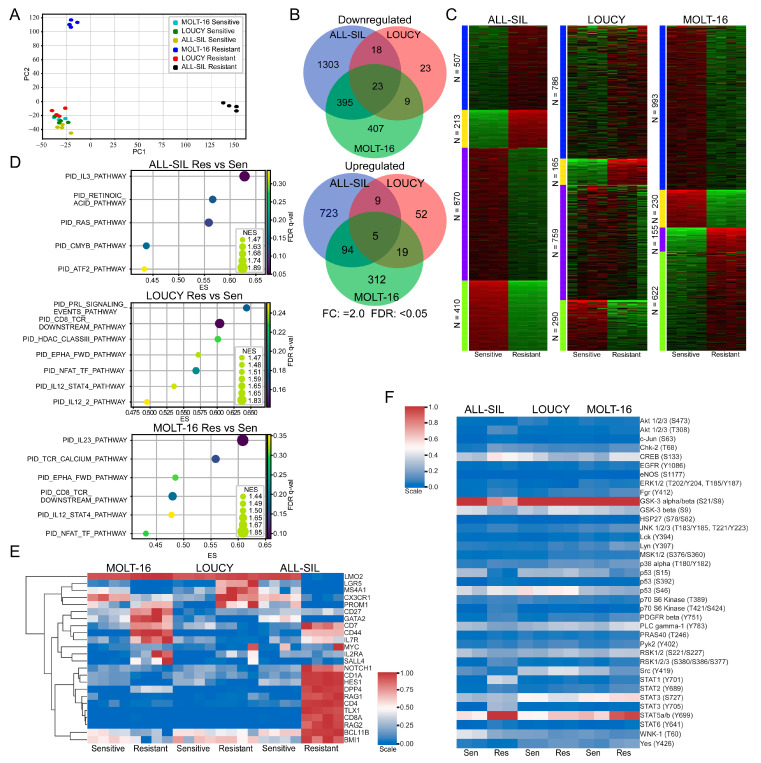
Venetoclax-resistant MOLT-16 and ALL-SIL display different clusters. (**A**) RNAseq data were used for principal component analysis (PCA) using Scikit-learn (sklearn.decomposition.PCA). (**B**) Differential gene expression in venetoclax-resistant cells was determined using the SciPy package, where genes displaying 2-fold or more upregulation or downregulation were recorded (FDR: <0.05). Venn diagram was generated using the web tool (http://bioinformatics.psb.ugent.be/webtools/Venn/ accessed on 1 March 2023). (**C**) k-Means clustering was used to determine clusters using the iDEP web application (http://bioinformatics.sdstate.edu/idep/ accessed on 1 March 2023). (**D**) Gene set enrichment in venetoclax-resistant cells compared to sensitive cells was determined using GSEA. (**E**) Expression data for known cancer stem cell markers were collected from RNAseq data. Clustermap from the Seaborn Python package was used to determine clusters. (**F**) Cells were lysed, and an equal amount of total protein was used to determine kinase phosphorylation.

Next, we isolated total mRNA from venetoclax-sensitive and venetoclax-resistant cells and analyzed them via RNA-seq. Samples were collected after being cultured in the absence of venetoclax for two weeks or longer. We observed that venetoclax-resistant ALL-SIL and MOLT-16 cells formed different clusters, while venetoclax-resistant LOUCY cells clustered with sensitive cell lines (Figure 4A). BCL2 family genes were also differentially regulated in different cells. For example, expression of *BAD*, *BIK,* and *BCL2* was downregulated more than two-fold in MOLT-16 (Supplementary Figure S4A), while expression of those genes remained unchanged or changed slightly in LOUCY (Supplementary Figure S4B) and ALL-SIL (Supplementary Figure S4C). The expression of *BMF*, *BCL2L11*, and *BCL2L1* was upregulated 2-fold or more, but *BAX* expression was downregulated 1.8-fold in ALL-SIL (Supplementary Figure S4C). While comparing sensitive and resistant cells, we observed cell line-specific transcriptional regulation (Figure 4B,C). Pathway enrichment analysis also showed differential enrichment in the pathways in different resistant cell lines (Figure 4D and Supplementary Figure S5). Furthermore, we observed the enrichment of different sets of cancer stem cell (CSC) markers in different cell lines (Figure 4E). Similarly, using the Proteome Profiler Human Phospho-Kinase Array Kit, we detected cell line-specific regulation of kinase activation (Figure 4F). However, we observed enrichment in cytokine signaling pathways in all three resistant cell lines (Figure 4D), which were also present in the phospho-kinase array, as we detected strong STAT5 phosphorylation in resistant cells (Figure 4F). 

## 3. Discussion

In this study, we generated venetoclax-resistant cell lines from the highly sensitive T-ALL cell lines ALL-SIL, LOUCY, and MOLT-16. We compared gene expression and protein kinase activation between sensitive and resistant cell lines using RNA sequencing and Proteome Profiler Human Phospho-Kinase Array. We observed the activation of cytokine signaling and cell line-specific enrichment of cancer stem cell markers.

The expression level of BCL2 varies during T-cell development, which is maintained in different subgroups of T-ALL. The expression level is relatively higher for ETP-ALL in comparison to non-ETP T-ALL patients. However, we observed higher levels of variation in BCL2 expression within non-ETP -TALL. T-ALL cell lines derived from patients of different ages (Supplementary Table S1) also showed different levels of BCL2 expression at the gene and protein levels and responded differentially to the BCL2 inhibitors. These observations suggest that the response to the BCL2 inhibitor is not limited to the BCL2 expression level. Expression of several other members including BCL-XL and MCL1 can modulate the efficacy of BCL2 inhibitors [21].

Expression of BCL2 and other anti-apoptotic BCL2 family proteins is tightly regulated by several signaling mediators. For example, T-ALL cells dependent on the JAK-family kinase TYK2 display elevated BCL2 levels, which can be suppressed by TYK2 depletion [32]. In mature T-ALL, TYK2-STAT1 signaling promotes survival through BCL2, which has also been linked to cytokine signaling [33]. Genetic abnormalities such as chromosomal rearrangement via translocation or deletion can drive elevated BCL2 expression in other diseases [34,35,36]. Thus, there are different mechanisms that contribute to elevated BCL2 expression, thereby aiding in cell proliferation and evading apoptosis. The identification of such genetic abnormalities in any of our venetoclax-resistant cells is another area to explore. However, we observed that T-ALL cells displaying venetoclax resistance exhibit differential expression of BCL2 family members, which does not always follow the classical BCL2/BCL2L1 expression ratio [25]. Furthermore, acquired resistance to venetoclax is probably achieved through the activation of several different pathways, including cytokine signaling, as we observed in GSEA, which is in line with the previous observation [33].

The signal transducer and activator of transcription (STAT) proteins are key transcription factors that play important roles in cytokine signaling [37]. We observed the enrichment of IL3, IL12, and IL23 signaling pathways and elevated phosphorylation of STAT5 and STAT3 in venetoclax-resistant cells. All three cytokines, IL3, IL12, and IL23, are known to activate STAT3 and STAT5 [38,39,40], further suggesting the link between venetoclax resistance and cytokine signaling in T-ALL. The role of STAT5 in venetoclax resistance has not been studied well. One study suggests that the inactivation of STAT5 enhances venetoclax efficacy [41]. Furthermore, the oncogenic mutant of type III receptor tyrosine kinase FLT3 (FLT3-ITD) activates STAT5 signaling and regulates BCL-XL and MCL1 expression [42,43,44]. Therefore, it is likely that venetoclax resistance through cytokine/STAT5 signaling is partially mediated through the transcriptional regulation of BCL2 family proteins. 

We observed enhanced expression of CSC markers in the venetoclax-resistant cells. CSCs are well known for their roles in therapy resistance [45,46,47,48]. For instance, the type I transmembrane protein CD44, which was upregulated in venetoclax-resistant MOLT-16 and ALL-SIL cells, was involved in the regulation of venetoclax sensitivity in acute myeloid leukemia [49]. Expression of CD44 was reported to be regulated by STAT5 activation in mastocytosis [50], possibly linking cytokine signaling to the expression of CSCs through STAT5 activation. Several other CSC markers which are upregulated in venetoclax resistance cells including NOTCH1 and IL7R have been reported to be involved in therapy resistance in different settings [51,52]. Taken together, our data suggest that venetoclax resistance in T-ALL can be mediated through the activation of cytokine signaling, which might eventually regulate the expression of cancer stem cell markers.

## 4. Methods and Materials

### 4.1. Cell Lines

The human leukemia cell lines (CCRF-CEM, CML-T1, DND-41, JURKAT, KE-37, MOLT-16, PF-382, P12-ICHIKAWA, and RPMI-8402) were grown in RPMI 1640 medium containing 10% heat-inactivated fetal bovine serum (FBS) from ThermoFisher Scientific, Waltham, MA, USA, 100 U/mL penicillin, and 100 µg/mL streptomycin from Corning, USA. The ALL-SIL, CTV-1, LOUCY, MOLT-4, and TALL-1 cell lines, on the other hand, were cultured in RPMI 1640 supplemented with 20% heat-inactivated FBS from ThermoFisher Scientific, Waltham, MA, USA, 100 U/mL penicillin and 100 µg/mL streptomycin from Corning, USA. All the cell lines were obtained from Deutsche Sammlung von Mikroorganismen und Zellkulturen (DSMZ, Braunschweig, Germany) and maintained in a ThermoFisher Scientific (Waltham, MA, USA) Heraeus BBD 6220 Incubator at 37 °C with 5% CO_2_. The cell lines were routinely screened for the presence of mycoplasma. 

### 4.2. Drug Sensitivity Assays

The viability of T-ALL cell lines was evaluated against different concentrations of BCL-2 inhibitors, navitoclax, and venetoclax, in 96-well plates by seeding the cells at a density of 20,000 cells per well. To determine the effective concentration 50 (EC_50_) for venetoclax, venetoclax-resistant cell lines (MOLT-16, LOUCY, and ALL-SIL) were seeded in the same way. After a 48-h incubation period, 10 µL of PrestoBlue was added to each well and incubated for 2 h. Fluorescence was then measured using a plate reader, and EC_50_ values were calculated using GraphPad Prism 5.0 software.

### 4.3. Western Blot and Antibodies

All cell lines were lysed in a RIPA buffer supplemented with protease/phosphatase inhibitors (PMSF, Trasylol, and Na_3_VO_4_). The bicinchoninic acid (BCA) assay method (ThermoFisher Scientific, Waltham, MA, USA) was used to determine the protein concentration of the total cell lysates. Around 10 µg of lysates were separated on SDS-PAGE gels; this was followed by their transfer to polyvinylidene difluoride (PVDF) membranes. The membranes were incubated with a panel of different primary antibodies. The anti-BCL2 (sc-509, 1:1000 dilution) and anti-β-actin-HRP (sc-47778, 1:2000 dilution) were obtained from Santa Cruz Biotechnology, Dallas, TX, USA. Anti-BCLXL (BCL2L1, 10783-1-AP, 1:4000) and anti-MCL1 (16225-1-AP, 1:2000) antibodies were from ProteinTech, Rosemont, IL, USA. For immunodetection, all the blots were incubated with the respective horseradish peroxidase-conjugated secondary antibodies, developed with the Luminata Forte Western HRP substrate (Millipore), and imaged with the Amersham Imager 600 (GE Healthcare, Danderyd, Sweden). ImageJ (NIH, Bethesda, MD, USA) was used to perform a densitometric analysis of the protein bands.

### 4.4. Real-Time Quantitative PCR

Total RNA was extracted from T-ALL cell lines and even venetoclax-resistant T-ALL cells, using the RNeasy mini kit (Qiagen) following the manufacturer’s instructions. The High-Capacity cDNA Reverse Transcription Kit (ThermoFisher Scientific, Waltham, MA, USA) was used to synthesize cDNA according to the manufacturer’s instructions. RT-qPCR was run to assess gene expression using gene-specific qPCR primer assays (ThermoFisher Scientific, Waltham, MA, USA) and an Applied Biosystems QuantStudio 7 Flex detection system. Each sample was analyzed in quadruplicate, and gene expression was normalized to the endogenous controls such as GAPDH and β-Actin. Relative changes in gene expression were calculated with the help of the comparative Ct method. Different probes used for qPCR included BCL2, BCL2L1, BCL2L2, MCL1, GAPDH, and β-Actin. These probes were ordered from ThermoFisher Scientific, Waltham, MA, USA. Target gene expression levels were normalized against GAPDH and β—actin, and relative expression was determined using the ΔΔCt method.

### 4.5. RNAseq Analysis

Total RNA was extracted from venetoclax-resistant cells using the RNeasy mini kit (Qiagen N.V. Venlo, The Netherlands) following the manufacturer’s instructions. The quality of total RNA was checked by Bioanalyzer (Agilent, Santa Clara, CA, USA), and the samples with an RNA integrity greater than 8 were further analyzed following the previously described method [53] with the help of the Center for Translational Genomics (CTG) at Lund University.

### 4.6. Phosphokinase Array

The Proteome Profiler Human Phospho-Kinase Array Kit (ARY003C) was obtained from R&D Systems (Minneapolis, MN, USA). Venetoclax-sensitive and venetoclax-resistant cells were lysed, and the lysates were processed according to the manufacturer’s protocol and also described previously [5,54].

### 4.7. Pharmacogenomic Data

Gene expression data corresponding to seven different datasets were downloaded from the NCBI Gene Expression Omnibus. The expression of some BCL-2 family genes in T-ALL patients was assessed in all the datasets, which also showed bifurcation into ETP and non-ETP groups. Moreover, IC_50_ data for a variety of BCL-2 family inhibitors impacting various T-ALL cell lines were downloaded from the Genomics of Drug Sensitivity in Cancer (GDSC).

### 4.8. Generating Venetoclax-Resistant Cells

Venetoclax-resistant T-ALL cell lines (MOLT-16, ALL-SIL, and LOUCY) were generated from their parental cells via the multistep exposure of cells to increasing concentrations of venetoclax, starting from 10 nM. The concentrations were doubled when the treated cells showed proliferation at an equal rate to the untreated parental cells. Venetoclax concentrations were increased at regular intervals until a 10 µM concentration was reached for MOLT-16 and ALL-SIL and a 5 µM concentration was reached for LOUCY cells. They were then checked for venetoclax resistance. These cells were further grown for two weeks in the absence of venetoclax. All the cells were retested for drug resistance before any further studies.

### 4.9. Gene Set Enrichment Analysis (GSEA)

Gene expression data of venetoclax-resistant and venetoclax-sensitive T-ALL cell lines were used to run Gene Set Enrichment Analysis (GSEA) using the GSEA 4.0.2 software (Broad Institute, Cambridge, MA, USA) with the Molecular Signatures database MSigDB to identify pathways enriched in venetoclax-resistant cells. 

### 4.10. Statistical Analysis

Statistical analysis was performed using the GraphPad Prism 5.0 (La Jolla, CA, USA) software, where data were expressed as mean ± SE. Unpaired Student’s *t*-test and one-way ANOVA with Bonferroni’s post-test were used where applicable. *p* ≤ 0.05 was considered significant.

## Data Availability

All raw data are available upon request.

## Supplementary Materials

The following supporting information can be downloaded at: https://www.mdpi.com/article/10.3390/ijms24055004/s1.
