# Supplementary material for: Venetoclax-Resistant T-ALL Cells Display Distinct Cancer Stem Cell Signatures and Enrichment of Cytokine Signaling
Source: Int J Mol Sci. 2023 Mar 5;24(5):5004. doi: 10.3390/ijms24055004 (PMC10003524; doi:10.3390/ijms24055004)
Supplement: Supplementary file 1 [file ijms-24-05004-s001.zip › ijms-2235102-supplementary.pdf]

Supplementary figures

***Supplementary figure S1:* T-ALL patients with both the ETP and non-ETP groups display differential expression of BCL2 family members.** Gene expression data for 7 T-ALL datasets, representing both the ETP and non-ETP groups were downloaded from the NCBI Gene Expression Omnibus. (A) Relative mRNA expression of BCL2, BCL2L1, BCL2L2, and MCL1 of T-ALL patients from the 7 datasets were plotted in dot plots. The red bar represents the SEM. (B) The covariance matrix for the BCL-2 family gene expression was calculated with the cov.wt function in R. (C) Expression values after the removal of ETP-ALL patients are presented. (D) The covariance matrix for the BCL-2 family gene expression after removal of ETP-ALL samples.

A

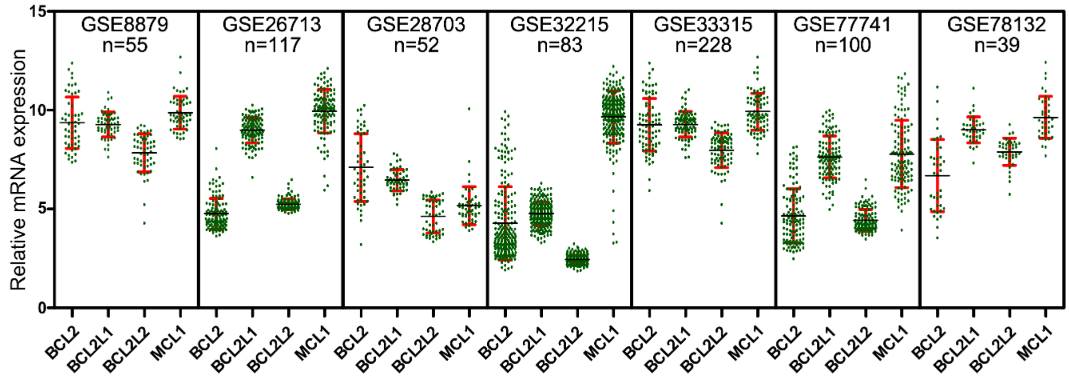

B

GSE8879	BCL2	BCL2L1	BCL2L2	MCL1
BCL2	1.69	-0.13	0.16	-0.01
BCL2L1	-0.13	0.41	0.03	0.07
BCL2L2	0.16	0.03	0.93	0.21
MCL1	-0.01	0.07	0.21	0.70

GSE26713	BCL2	BCL2L1	BCL2L2	MCL1
BCL2	0.61	0.02	-0.03	-0.06
BCL2L1	0.02	0.40	-0.06	0.14
BCL2L2	-0.03	-0.06	0.07	-0.09
MCL1	-0.06	0.14	-0.09	1.23

GSE28703	BCL2	BCL2L1	BCL2L2	MCL1
BCL2	2.92	-0.04	0.81	0.33
BCL2L1	-0.04	0.29	-0.10	0.00
BCL2L2	0.81	-0.10	0.68	0.06
MCL1	0.33	0.00	0.06	0.93

GSE32215	BCL2	BCL2L1	BCL2L2	MCL1
BCL2	3.44	-0.10	-0.05	-0.11
BCL2L1	-0.10	0.36	-0.03	0.05
BCL2L2	-0.05	-0.03	0.05	-0.04
MCL1	-0.11	0.05	-0.04	1.75

GSE33315	BCL2	BCL2L1	BCL2L2	MCL1
BCL2	1.76	-0.06	0.12	-0.15
BCL2L1	-0.06	0.41	0.03	0.11
BCL2L2	0.12	0.03	0.77	0.23
MCL1	-0.15	0.11	0.23	0.86

GSE77741	BCL2	BCL2L1	BCL2L2	MCL1
BCL2	2.21	-0.05	-0.09	0.18
BCL2L1	-0.05	0.69	-0.08	0.25
BCL2L2	-0.09	-0.08	0.10	-0.10
MCL1	0.18	0.25	-0.10	1.43

GSE78132	BCL2	BCL2L1	BCL2L2	MCL1
BCL2	3.33	-0.33	0.35	-0.17
BCL2L1	-0.33	0.43	-0.10	0.03
BCL2L2	0.35	-0.10	0.47	0.16
MCL1	-0.17	0.03	0.16	1.13

C

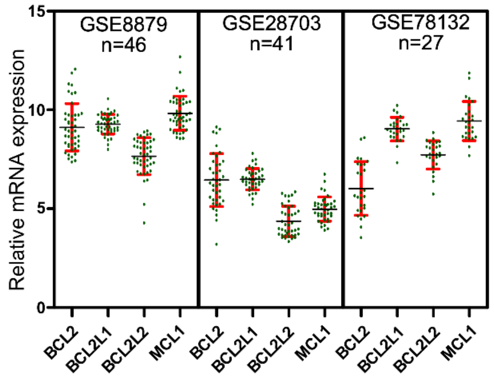

D

GSE8879	BCL2	BCL2L1	BCL2L2	MCL1
BCL2	1.43	-0.04	-0.10	0.01
BCL2L1	-0.04	0.25	0.07	0.03
BCL2L2	-0.10	0.07	0.87	0.19
MCL1	0.01	0.03	0.19	0.74

GSE28703	BCL2	BCL2L1	BCL2L2	MCL1
BCL2	1.80	0.09	0.31	0.12
BCL2L1	0.09	0.30	-0.10	0.05
BCL2L2	0.31	-0.10	0.59	-0.09
MCL1	0.12	0.05	-0.09	0.38

GSE78132	BCL2	BCL2L1	BCL2L2	MCL1
BCL2	1.84	-0.38	0.37	-0.42
BCL2L1	-0.38	0.35	-0.10	-0.08
BCL2L2	0.37	-0.10	0.51	0.07
MCL1	-0.42	-0.08	0.07	0.97

***Supplementary figure S2:* T-ALL cell lines display a differential response to BCL2 inhibitors.** (A)BCL2/BCL2L1 ratios presented in figure 1C were reanalyzed for ALL-SIL, LOUCY, and MOLT-16 cell lines. One-way ANOVA with multiple comparisons was used to determine p values. (B-E) IC_{50} data for BCL2 family inhibitors were downloaded from the Genomics of Drug Sensitivity in Cancer (GDSC). Red dots represent T-ALL cell lines. IC_{50} values of (B) BCL-2 and BCL-XL specific inhibitors; (C) BCL-2 inhibitors targeting two BCL-2 family members other than BCL-2; (D) BCL-2 inhibitors targeting three to four other BCL-2 family members other than BCL-2; and (E) MCL-1 specific inhibitors, were plotted for various T-ALL cell lines.

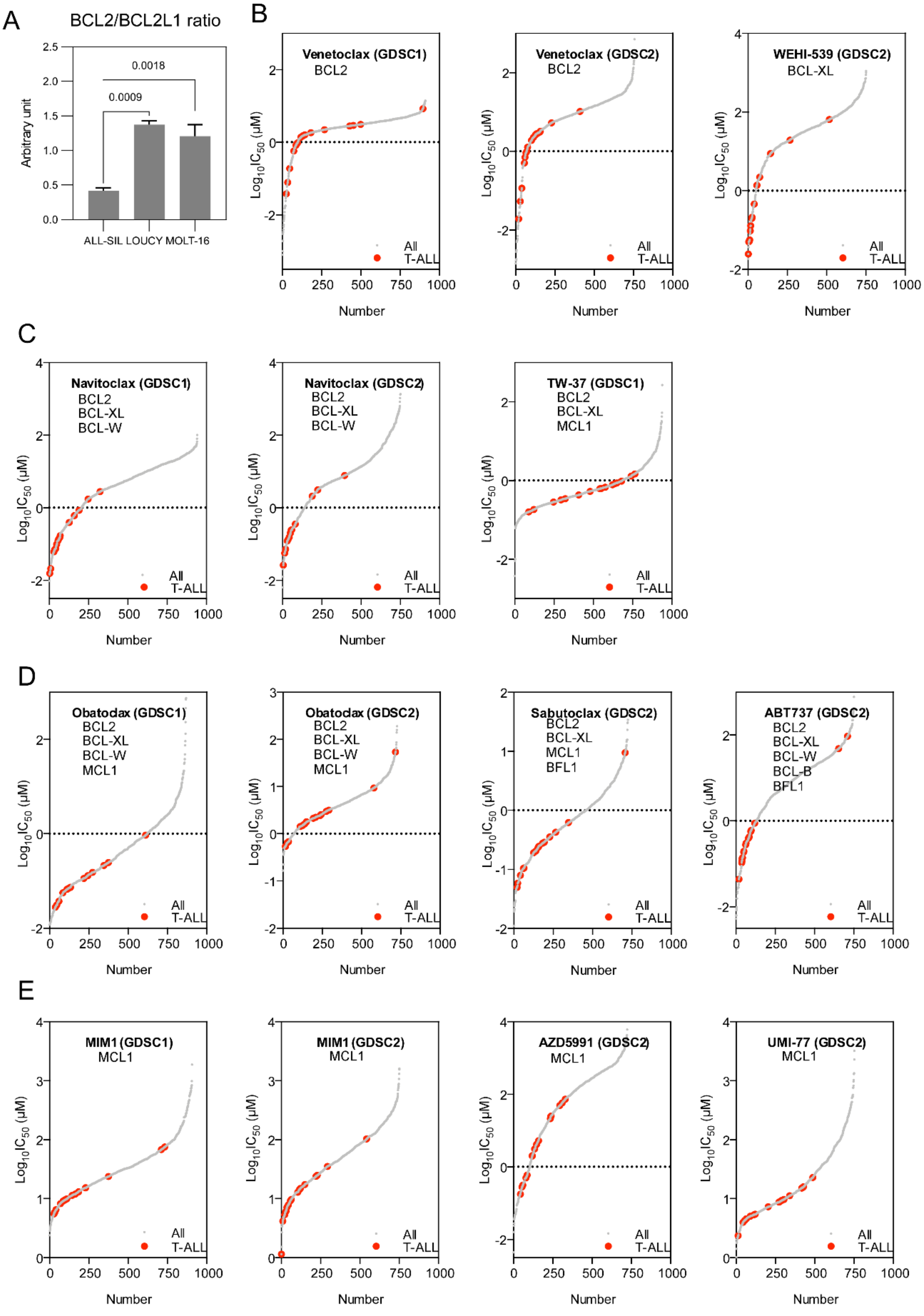

Supplementary figure S3: Relative BCL2 expression for MOLT-16, ALL-SIL, and LOUCY cell lines presented in figure 3D was analyzed using one-way ANOVA with multiple comparisons to determine p values.

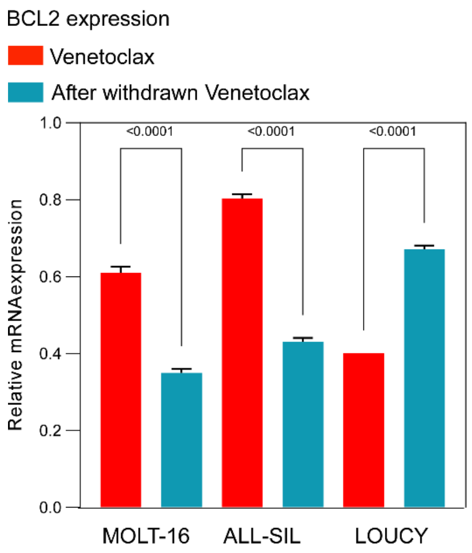

Supplementary figure S4: **BCL2 family gene expression in venetoclax-sensitive and -resistant cells.** (A) MOLT-16, (B) LOUCY, and (C) ALL-SIL cells, both venetoclax-sensitive/resistant were subjected to RNASeq, and the gene expression data of various BCL2 family members are presented here.

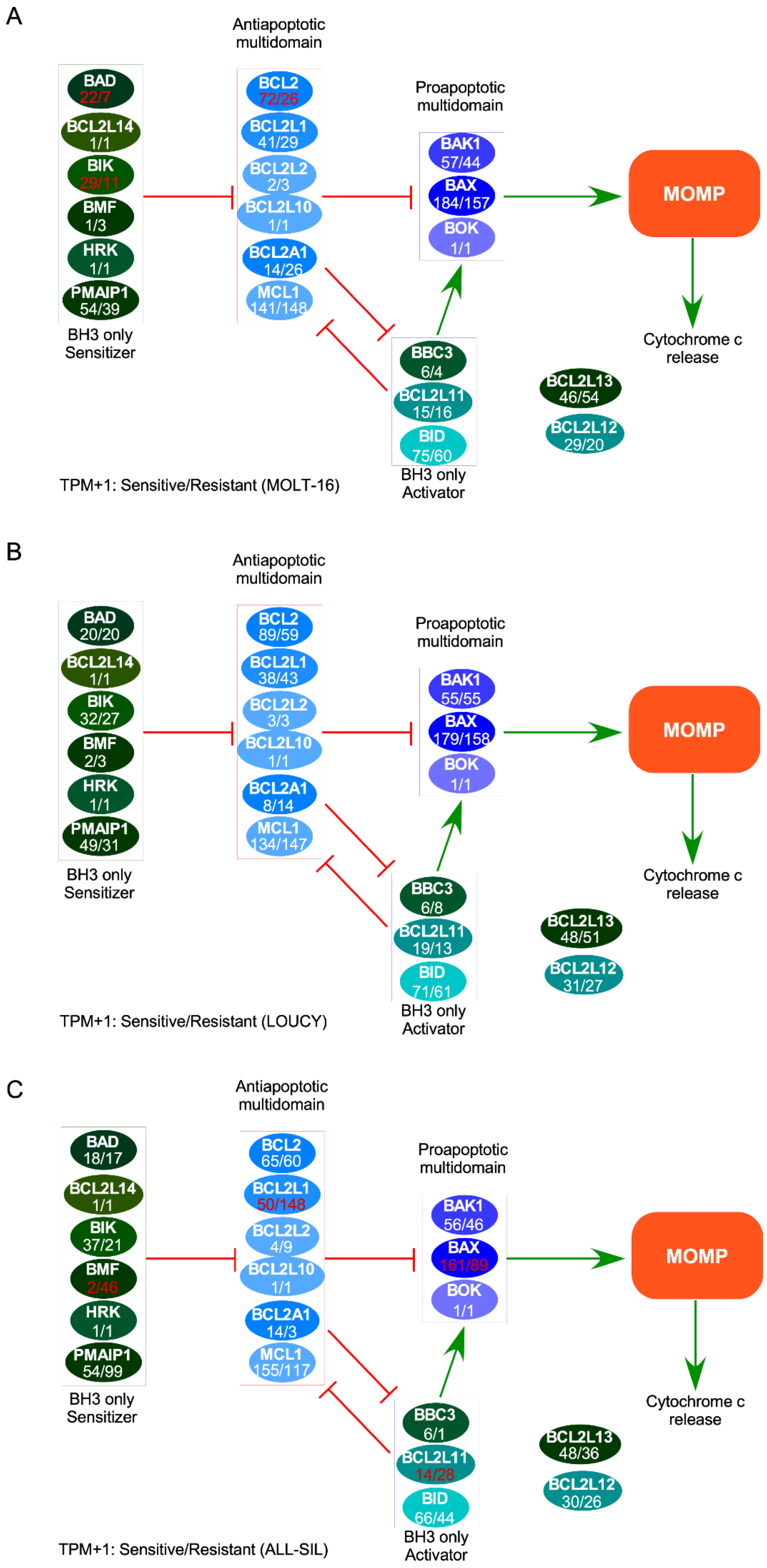

Supplementary figure S5: **Gene set enrichment analysis in venetoclax-resistant cells.** Gene set enrichment in venetoclax-resistant (A) ALL-SIL, (B) LOUCY and (C) MOLT-16 was determined by GSEA using the Molecular Signatures database MSigDB.

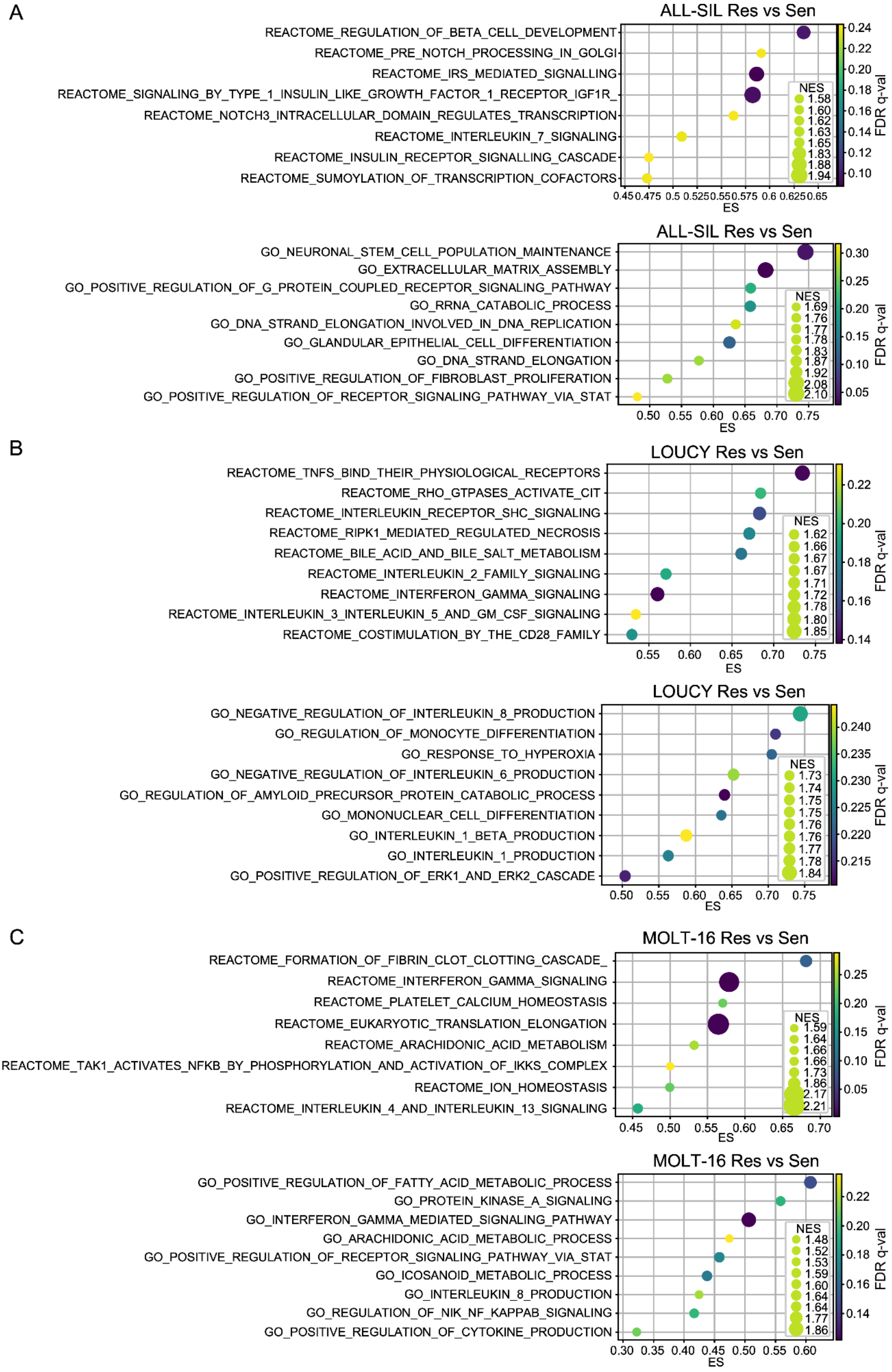

Supplementary Table

***Supplementary Table S1*: T-ALL cell lines**

Cell line	Gender (Age)	Maturity	Mutation	Expression
ALL-SIL	Male (17)	T-ALL relapse	NUP214-ABL1	TLX1
CCRF-CEM	Female (3)	T-ALL relapse		NKX2-5
CML-T1	Female (36)	CML patient	BCR-ABL1	
CTV-1	Man (40)	AML patient	TAL1-TRB	
DND-41	Male (13)	T-ALL; type III cortical	CDKN2A, CDKN2B deletion, p53 mutation	TLX3
JURKAT	Male (14)	ALL relapse		
KE-37	Male (27)	ALL		
LOUCY	Female (38)	T-ALL FAB L2 Chemo-resistant	SET-NUP214	MEF2C
MOLT-4	Male (19)	ALL relapse		
MOLT-16	Female (5)	T-ALL relapse		
P12-ICHIKAWA	Male (7)	ALL		
PF-382	Female (6)	ALL		
RPMI-8402	Female (16)	ALL		
TALL-1	Male (28)	Lymphosarcoma		
